# From image processing to computational neuroscience: a neural model based on histogram equalization

**DOI:** 10.3389/fncom.2014.00071

**Published:** 2014-07-17

**Authors:** Marcelo Bertalmío

**Affiliations:** Department of Information and Communication Technologies, Universitat Pompeu FabraBarcelona, Spain

**Keywords:** neural model, Wilson-Cowan equation, efficient coding, redundancy reduction, contrast enhancement, lightness induction

## Abstract

There are many ways in which the human visual system works to reduce the inherent redundancy of the visual information in natural scenes, coding it in an efficient way. The non-linear response curves of photoreceptors and the spatial organization of the receptive fields of visual neurons both work toward this goal of efficient coding. A related, very important aspect is that of the existence of post-retinal mechanisms for contrast enhancement that compensate for the blurring produced in early stages of the visual process. And alongside mechanisms for coding and wiring efficiency, there is neural activity in the human visual cortex that correlates with the perceptual phenomenon of lightness induction. In this paper we propose a neural model that is derived from an image processing technique for histogram equalization, and that is able to deal with all the aspects just mentioned: this new model is able to predict lightness induction phenomena, and improves the efficiency of the representation by flattening both the histogram and the power spectrum of the image signal.

## 1. Introduction

The human visual system works in many ways in order to efficiently encode the visual information coming from natural environments, reducing its inherent redundancy, as proposed in the seminal work of Barlow (1961) (see Olshausen and Field, 2000 for a review). For instance, while natural scenes have luminance distributions which are very lopsided, with a high peak and a very rapid fall-off, photoreceptors encode this information with signals that have a much more even distribution: indeed, photoreceptors perform histogram equalization, as demonstrated by Laughlin (1981). And the receptive fields of visual neurons, both retinal and post-retinal, compensate the 1/*f*^2^ decay of the power spectrum of natural images, whitening the spectrum of the resulting signal and thus minimizing interpixel redundancies and increasing coding efficiency (see Atick, 1992; Dan et al., 1996 where the existence of whitening at the local geniculate nucleus is demonstrated for natural images).

Apart from efficiency in coding, another very important aspect is that of biological efficiency in terms of *wiring*. The resolution of retinal mosaics is limited by the number of axons that can pass through the optic nerve, which acts as a bottleneck (Olshausen, 2003). But the visual system is able to achieve a visual acuity beyond the limit imposed by the number of photoreceptors at the retina: in their classical paper on contrast constancy, Georgeson and Sullivan (1975) suggest that there are cortical mechanisms for contrast enhancement that compensate for the blurring produced in early stages of the visual process. Very recently Martinez et al. (2014) have confirmed that contrast enhancement takes place at the lateral geniculate nucleus (LGN) and, remarkably, the authors point out that this contrast enhancement procedure is very much alike the common techniques used in image processing.

Alongside mechanisms for coding and wiring efficiency, there is neural activity in region *V*1 of the human visual cortex that correlates with the perceptual phenomenon of lightness induction, as proven by Pereverzeva and Murray (2008). The term *lightness induction* or *achromatic induction* designates the visual phenomenon by which the perceived reflectance of an object depends on its surround. It can take the form of *lightness contrast*, when the object's lightness shifts away from that of its surroundings: a dark object on a light background appears even darker, or a light object in a dark surround becomes even lighter. The reverse is called *lightness assimilation*, in which case the appearance of the object shifts in the direction of the lightness of its surround. As pointed out by Shevell (2003), lightness assimilation occurs in situations of high spatial frequency while lightness contrast is associated with relatively lower spatial frequencies.

Our contribution in this paper is to propose a neural activity model, a partial differential equation (PDE) in the form of a Wilson-Cowan equation (Wilson and Cowan, 1972), which takes care simultaneously of the four aspects mentioned above: it performs histogram equalization, spectrum whitening, contrast enhancement, and it also predicts lightness induction. The proposed model is based on a state of the art method for color and contrast enhancement from the image processing literature, so we start the following section reviewing some key image processing concepts.

## 2. Image processing for contrast enhancement

### 2.1. Histogram equalization

**Figure 1:**
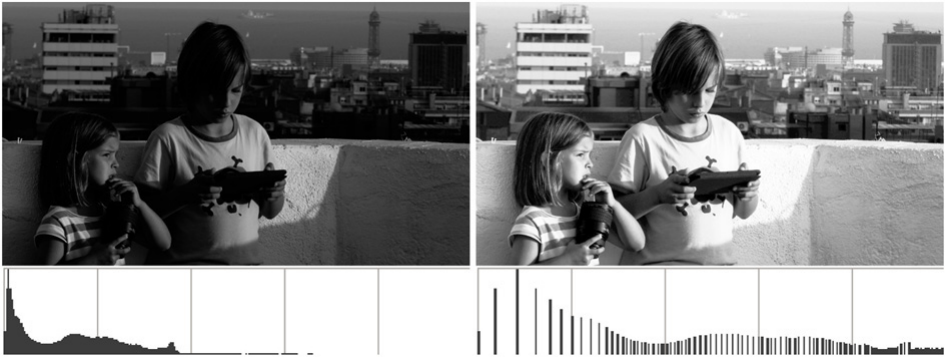
**Left:** image and associated histogram. **Right:** after histogram equalization.

Histogram equalization is a classical, very basic image processing technique dating at least to the early 1970s (see Pratt, 2007 and references therein), aiming at enhancing the contrast and improving the appearance of images by way of re-distributing their levels uniformly accross the available range. In this sense, an image would be optimal if its histogram were flat or “equalized,” meaning that all the range is used and all levels are represented by the same amount of pixels. Therefore, when an image has a flat histogram its cumulative histogram is simply a ramp, and this allows for a very straightforward computation for the histogram equalization procedure: assuming we are working on a graylevel image in the range [0,1], we have to substitute each level *g* in the original image by the value of its normalized cumulative histogram, *H*(*g*). The solution is computed very fast using a look-up table (LUT). An example result can be seen in Figure 1 (notice that, while the range has been expanded and the resulting image has a more even histogram, it's not actually uniform).

**Figure 2:**
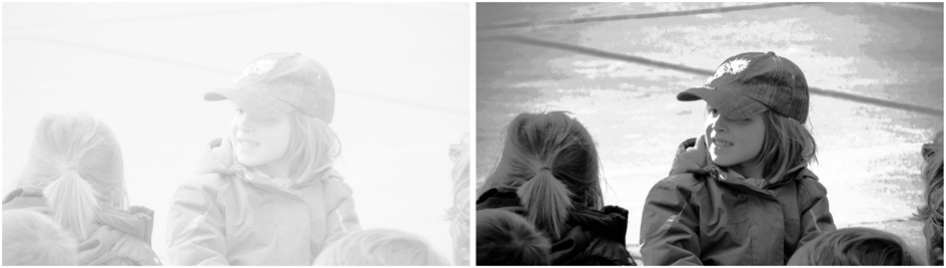
**Left:** original image. **Right:** after histogram equalization.

While in Figure 1 histogram equalization improves the visual appearance of the image, Figure 2 shows an example where the image is actually made worse, which Pratt (2007) points out is often the case when the image is overexposed, as it is here. This is aggravated by the fact that the equalization procedure is a one-shot technique, that only produces a final result, without any “in-between,” so if the resulting image shows any type of unpleasant artifact there is nothing to do about it. This issue was addressed by Sapiro and Caselles (1997), who proved that the minimization of the energy functional

(1) $$E(I)=2\sum_{x}{\left(I(x)-\frac{1}{2}\right)}^{2}-\frac{1}{AB}\sum_{x}\sum_{y}|I(x)-I(y)|$$

produces an image *I* with a flat histogram. The range of *I* is [0,1], *x*, *y* are pixels and *A*, *B* are the image dimensions. While the result of histogram equalization is very often unsatisfactory and can't be altered, Sapiro and Caselles (1997) propose to start with an input image *I*_0_ and apply to it step after step of the minimization of Equation 1, letting the user decide when to stop. If the user lets the minimization run to convergence, she'll get the same result as with a LUT, but otherwise a better result can be obtained if the iterative procedure stops before the appearance of severe artifacts. The squared differences in the first term of Equation 1 and the absolute differences in the second one are required to ensure that the minimization yields an image with equalized histogram, see Sapiro and Caselles (1997) for details. The energy in Equation 1 can be interpreted as the difference between two positive and competing terms,

(2) E(I)=D(I)−C(I).

The first term measures the dispersion around the average value of ½, as in the *gray world* hypothesis for color constancy, stating that our visual system estimates the illuminant as one half the average of the colors of the scene, an observation made by Judd (1940, 1979a) and formalized by Buchsbaum (1980). The second term measures the contrast as the sum of the absolute value of the pixel differences.

### 2.2. Perceptually-based contrast enhancement

The abovementioned measure of contrast is global, not local, i.e., the differences are computed regardless of the spatial locations of the pixels. This is not consistent with how we *perceive* contrast, which is in a localized manner, at each point having neighbors exert a higher influence than far-away points. Using the concepts introduced by the popular perceptually-based color correction method ACE of Rizzi et al. (2003), the authors of Bertalmío et al. (2007) propose an adapted version of the functional of Equation 1 that complies with some very basic visual perception principles, namely those of locality, color constancy and *white patch* (the latter stating that the brightest spot in the image is perceived as white, an observation that is often attributed, incorrectly, to the Retinex theory of Land (1977), but which has a long history that dates back at least to the works of Helmholtz, as explained by Judd (1979b,c)):

(3) $$\begin{array}{l} E(I)=\frac{\alpha }{2}\sum_{x}{\left(I(x)-\frac{1}{2}\right)}^{2}-\gamma \sum_{x}\sum_{y}w(x,y)|I(x)\\ \;\;\;\;\;\;\;\;\;\;\;\;-I(y)|+\frac{\beta }{2}\underset{x}{\sum }{\left(I(x)-I_{0}(x)\right)}^{2}, \end{array}$$

where *w* is a distance function such that its value decreases as the distance between *x* and *y* increases, *I*_0_ is the original image and α, β and γ are positive weights (which can be chosen so as to guarantee the white patch property, see Bertalmío et al., 2007 for details). The gradient descent equation for the functional in Equation 3 is the following, and its numerical implementation is essentially equivalent to the method of Rizzi et al. (2003):

(4) $$\begin{array}{l} I_{t}(x)=-\alpha \left(I(x)-\frac{1}{2}\right)+\gamma \sum_{y}w(x,y)sgn(I(x)-I(y))\\ \;-\beta (I(x)-I_{0}(x)). \end{array}$$

Starting from *I* = *I*_0_, we iterate Equation 4 until we reach a steady state, which will be the result of this algorithm.

By minimizing the energy in Equation 3 we are locally enhancing contrast (second term) and promoting color constancy by discounting the illuminant (first term), while preventing the image from departing too much from its original values (third term). We could also say that the minimization of Equation 3 approximates *local* histogram equalization.

The method of Bertalmío et al. (2007) has several good properties:

1. It yields very good color constancy results, being able to remove strong color casts and to deal with non-uniform illumination (a challenging scenario for most color constancy algorithms, see Bertalmío, 2014).
2. It increases the dynamic range of the image (i.e., it tends to “flatten” its histogram).
3. It has a very good local contrast enhancement performance, producing results without halos, spurious colors or any other kind of visual artifact.
4. It can deal with both underexposed and overexposed pictures.
5. It reproduces visual perception phenomena such as simultaneous contrast and the Mach Band effect.

But regarding color constancy, there is also a very interesting and close connection with the classical approach of Retinex. In their kernel-based Retinex (KBR) formulation, Bertalmío et al. (2009) take all the essential elements of the Retinex theory of Land (1977) (channel independence, the ratio reset mechanism, local averages, non-linear correction) and propose an implementation that is intrinsically 2D, and therefore free of the issues associated with the 1D paths used in the original Retinex algorithm. The results obtained with this algorithm comply with all the expected properties of Retinex (such as performing color constancy while being unable to deal with overexposed images) but don't suffer from the usual shortcomings such as sensitivity to noise, appearance of halos, etc. In Bertalmío et al. (2009) it is proven that there isn't any energy that is minimized by the iterative application of the KBR algorithm, and this fact is linked to its limitations regarding overxposed pictures. Using the analysis of contrast performed by Palma-Amestoy et al. (2009), Bertalmío et al. (2009) are able to determine how to modify the basic KBR equation so that it can also handle overexposed images, and the resulting, modified KBR equation turns out to be essentially the gradient descent of the energy given by Equation 3. In other words, the method of Bertalmío et al. (2007) can be seen as an iterative application of Retinex, although in a modified version that allows to produce good results also in the case of overexposed images.

## 3. A new neural model

### 3.1. Connection with neuroscience

The activity of a population of neurons in the region *V*1 of the visual cortex evolves in time according to the Wilson-Cowan equations (see Wilson and Cowan, 1972, 1973; Bressloff et al., 2002). Treating *V*1 as a planar sheet of nervous tissue, the state *a*(*r*, ϕ, *t*) of a population of cells with cortical space coordenates *r* ∈ ℝ^2^ and orientation preference ϕ ∈ [0, π) can be modeled with the following PDE (Bressloff et al., 2002):

(5) $$\begin{array}{l} \frac{\partial a(r,\phi ,t)}{\partial t}=-\alpha a(r,\phi ,t)\\ \;+\mu \int_{0}^{\pi }\int_{{\mathbb{R}}^{2}}\omega (r,\phi \|r^{\prime },\phi^{\prime })\sigma (a(r^{\prime },\phi^{\prime },t))dr^{\prime }d\phi^{\prime }\\ \;+h(r,\phi ,t), \end{array}$$

where α, μ are coupling coefficients, *h*(*r*, ϕ, *t*) is the external input (visual stimuli), ω(*r*, ϕ||*r*′, ϕ′) is a kernel that decays with the differences |*r* − *r*′|, |ϕ − ϕ′| and σ is a sigmoid function. If we ignore the orientation ϕ and assume that the input *h* is constant in time, it can be shown that Equation 5 is closely related to the gradient descent Equation 3, where neural activity *a* plays the role of image value *I*, sigmoid function σ behaves as the derivative of the absolute value function, and the visual input *h* is the initial image *I*_0_. This connection was already pointed out by Bertalmío et al. (2007), and Bertalmío and Cowan (2009) use it to argue that the Wilson-Cowan equations could therefore be the gradient descent of a certain energy, and also that there would appear to be a physical substrate at the cortex for the Retinex theory.

### 3.2. Lightness induction

**Figure 3:**
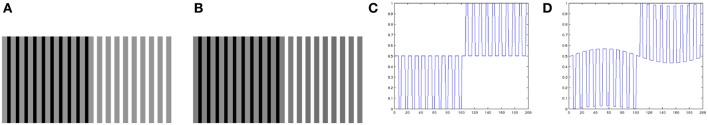
**(A)** Original image, example of lightness assimilation: the gray bars have all the same value but appear different over black and white backgrounds. **(B)** Result of applying the model of Bertalmío et al. (2007) to image **(A)**. **(C)** Profile of a line from image **(A)**. **(D)** Profile of a line from image **(B)**: notice how the model of Bertalmío et al. (2007) actually emulates lightness contrast rather than assimilation.

Looking closely at Equation 4, we can see that the spatial arrangement of the image data plays no role in it. Therefore, we can expect that the local contrast enhancement procedure of Bertalmío et al. (2007) will always produce lightness contrast, not assimilation, since as we mentioned earlier assimilation is linked to high spatial frequencies (Shevell, 2003). Figure 3 confirms this: (Figure 3A) produces lightness assimilation, because all gray bars have the same value but they are perceived darker when surrounded by black and lighter when surrounded by white; on the other hand, the result produced by Bertalmío et al. (2007) in Figure 3B actually emulates lightness contrast rather than assimilation, as the line profiles in Figures 3C,D show.

**Figure 4:**
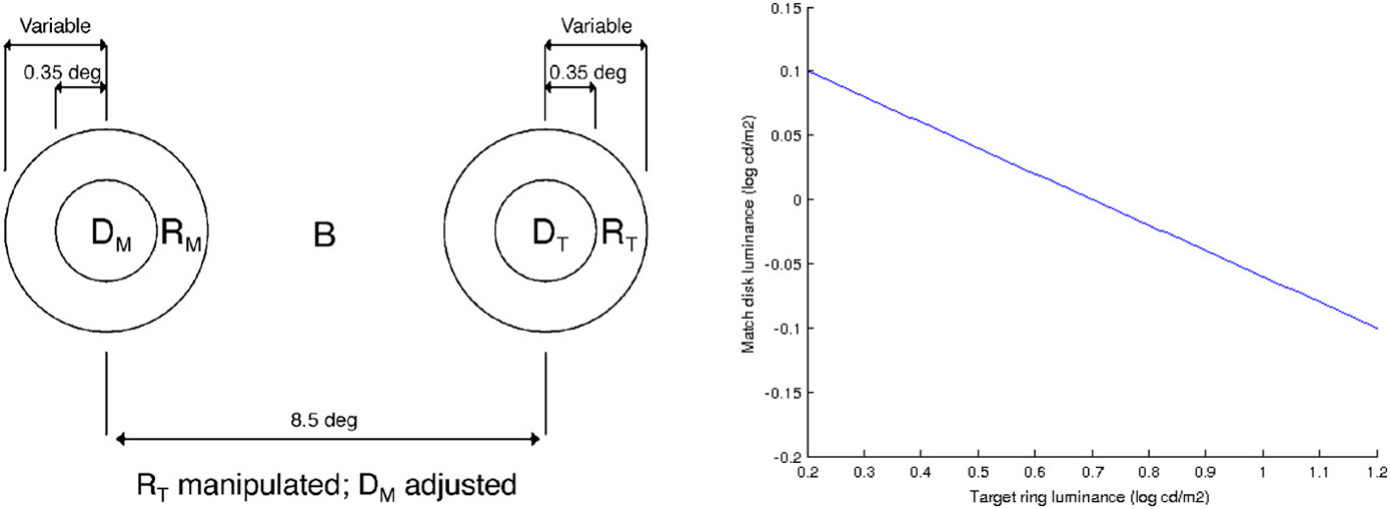
**Left:** diagram of the disk-and-ring display used by Rudd (2010), taken from that paper. **Right:** value of *D*_*M*_ as a function of *R*_*T*_, predicted with the model of Bertalmío et al. (2007).

**Figure 5:**
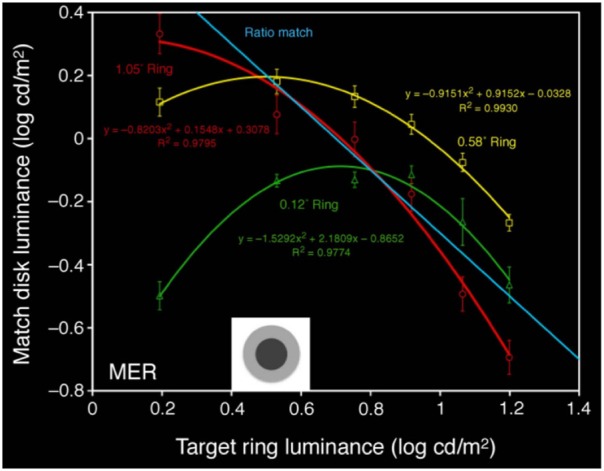
**Value of *D*_*M*_ as a function of *R*_*T*_: results of perceptual matches for different ring widths, from Rudd (2010)**.

Rudd (2010) studies lightness induction using a disk-and-ring (DAR) display for matching experiments, see Figure 4 (left). The intensity of the background *B*, of the left ring *R*_*M*_ and of the disk on the right *D*_*T*_ is kept constant; the intensity of the right ring *R*_*T*_ is modified, and the observer has to adjust the intensity of the left disk *D*_*M*_ so as to match the appearance of the right disk *D*_*T*_. Using the model of Bertalmío et al. (2007), the predicted value of *D*_*M*_ as a function of the varying *R*_*T*_ can be computed, and it is shown in Figure 4 (right). We can see that as *R*_*T*_ increases *D*_*M*_ always decreases, so according to this model we should only have lightness contrast in this situation. But the data from the perceptual experiments of Rudd (2010) says otherwise, see Figure 5: as *R*_*T*_ increases *D*_*M*_ also increases (lightness assimilation) until *R*_*T*_ reaches some value, beyond which *D*_*M*_ decreases (lightness contrast). These plots are well approximated by parabolas and, as the ring widths become larger, the resulting parabolas have their curvature decrease, implying that “assimilation is more likely to be observed with narrow surrounds” (Rudd, 2010).

### 3.3. Proposed model

In order to overcome the intrinsic limitations of Bertalmío et al. (2007) with respect to lightness induction, we should introduce spatial frequency in the energy functional. We propose a new model consisting in the following PDE, a modification of the gradient descent Equation (4):

(6) $$\begin{array}{l} I_{t}(x)=-\alpha (I(x)-\mu (x))+\gamma \left((1+{\left(\sigma (x)\right)}^{c}\right)\sum_{y}w(x,y)sgn(I(x)\\ \;-I(y))-\beta (I(x)-I_{0}(x)), \end{array}$$

where μ(*x*) is the mean average of the original image data computed over a neighborhood of *x*, σ(*x*) is the standard deviation of the image data computed over a small neighborhood of *x*, and the exponent *c* is a positive constant. The differences with respect to Equation 4 are that now the average in the first term is no longer global (the 1/2 value of Equation 4) but local, and that the weight for the second term is no longer a constant, but it changes both spatially and with each iteration, according to the local standard deviation σ: if the neighborhood over which it is computed is sufficiently small, standard deviation can provide a simple estimate of spatial frequency. But also, the standard deviation is commonly used in the vision literature as an estimate of local contrast. We have this contrast σ(*x*) raised to a power *c*, and this is also the case with other neural models where a power law is applied to the contrast, as we will briefly discuss later.

Again, this is a Wilson-Cowan type of neural activity model, where *I*_0_ is the visual input. We take *I*_0_ as a non-linear modification of the radiance stimulus, e.g., *I*_0_ could be the result of applying the Naka-Rushton equation, which models photoreceptor responses (see Shapley and Enroth-Cugell, 1984), to the radiance stimuli. As we did with Equation 4, we start with an image *I* = *I*_0_ and iterate Equation 6 until convergence, obtaining a result which we'll see is able to predict perceptual phenomena as well as improve the efficiency of the representation.

## 4. The proposed model predicts induction and improves efficiency

### 4.1. Predicting lightness induction

**Figure 6:**
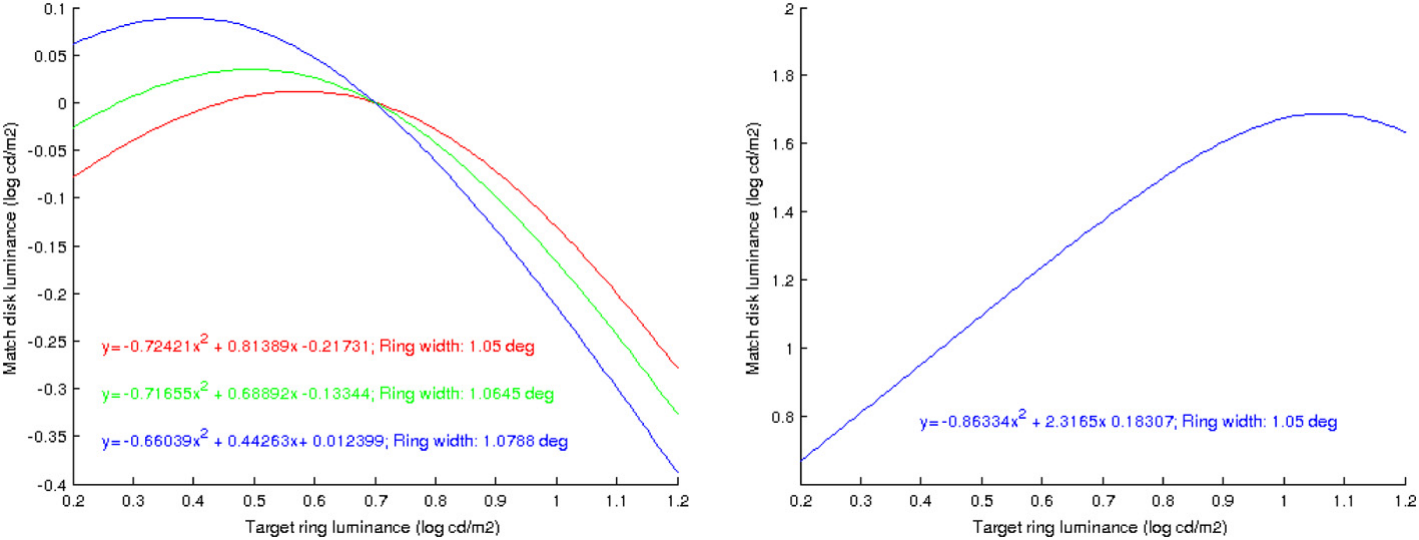
**Left:** predicted value of *D*_*M*_ as a function of *R*_*T*_, using the proposed model, for different ring widths; the plots are well approximated by parabolas, whose curvature decreases as the ring width increases. **Right:** prediction when the disk is a luminance increment with respect to the ring; the sign of the curvature remains negative.

Using this new model, now we can qualitatively predict the results of Rudd (2010). We fix *B*, *D*_*T*_, *R*_*M*_ and for each value of *R*_*T*_ we find the steady state of Equation 6 at the center of the right disk, at the middle of the right ring and at the middle of the left ring: these would be our model's predictions of the perceived values for *D*_*T*_, *R*_*T*_ and *R*_*M*_. Next, we compute the difference between the first two values and add it to the third, yielding the prediction of the perceived (lightness) value for *D*_*M*_, from which we can recover the actual luminance value *D*_*M*_ using again Equation 6 (see Appendix for implementation details). From these results we can derive the following conclusions, that corroborate the findings of the perceptual experiments of Rudd (2010):

- As shown in Figure 6 (left), the predicted match luminance plots are no longer linear but quadratic, with an initial lightness assimilation regime for low values of *R*_*T*_ followed by a lightness contrast part.
- The curvature of these parabolas decreases with increasing ring width.
- The previous experiments were for a *double-decrement* display: *B* > *R*_*T*_ > *D*_*T*_. If we now make the ring have a value in between disk and background, *B* > *R*_*T*_ < *D*_*T*_, the plot curvature remains negative as in the *double-decrement* case, see Figure 6 (right). As reported by Rudd (2010), this behavior can not be predicted with some other neural models, like that of Rudd and Arrington (2001), with which the curvature changes sign in this situation.

**Figure 7:**
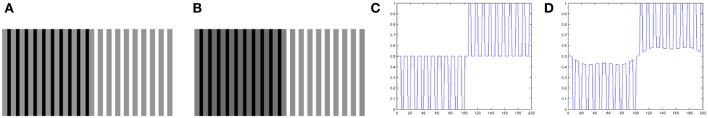
**(A)** Original image, example of lightness assimilation: the gray bars have all the same value but appear different over black and white backgrounds. **(B)** Result of applying the proposed model to image **(A)**. **(C)** Profile of a line from image **(A)**. **(D)** Profile of a line from image **(B)**: notice how the proposed model is capable of emulating lightness assimilation.

We can also predict lightness assimilation in the previous example of the alternating gray bars of Figure 3, as we now show in Figure 7.

**Figure 8:**
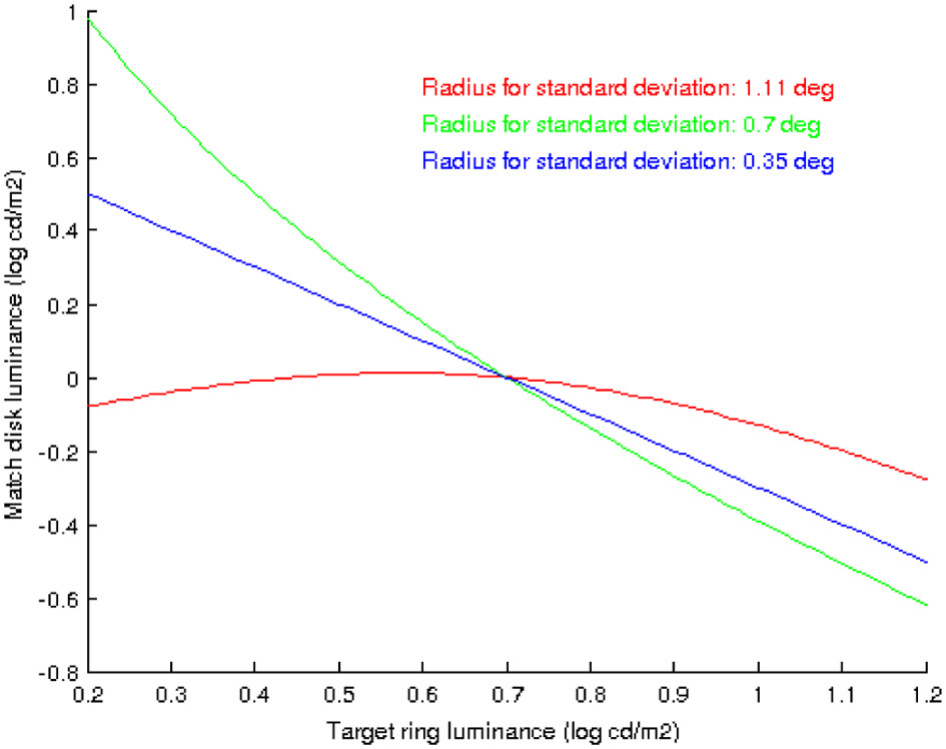
**The shape of the curves that predict the value of *D*_*M*_ as a function of *R*_*T*_, using the proposed model, depend on the extent of the neighborhood over which the standard deviation is computed**. In red: the neighborhood covers disk, ring and some background. In green: the neighborhood covers disk and ring but no background. In blue: the neighborhood covers just the disk.

It is interesting to note that the shape of the curves in Figure 6 does vary with extent of the neighborhood over which the standard deviation is computed, as Figure 8 shows: when the neighborhood covers disk, ring and some background we have an inverted parabola as before (red curve), but if we decrease the neighborhood size so that it only covers disk and ring but no background then the parabola concavity is reversed (green curve), and if the neighborhood is further reduced so that it only covers the disk then the curve is no longer parabolic but linear.

Finally, we may point out that some recent models which also predict lightness induction based on neural attributes of the visual system can be found in Otazu et al. (2008) and Penacchio et al. (2013).

### 4.2. Efficiency: redundancy reduction and contrast enhancement

In this section we argue that the proposed model of Equation 6, which as we have seen has the form of a Wilson-Cowan equation, performs local contrast enhancement and is closely related to basic image processing techniques, is a good candidate for a neural model providing the contrast constancy effects described by Georgeson and Sullivan (1975) and Martinez et al. (2014). But furthermore we will now see how this new neural model, applied to signals already encoded by photoreceptors, further improves efficiency by reducing redundancy: flattening the histogram and whitening the power spectrum.

**Figure 9:**
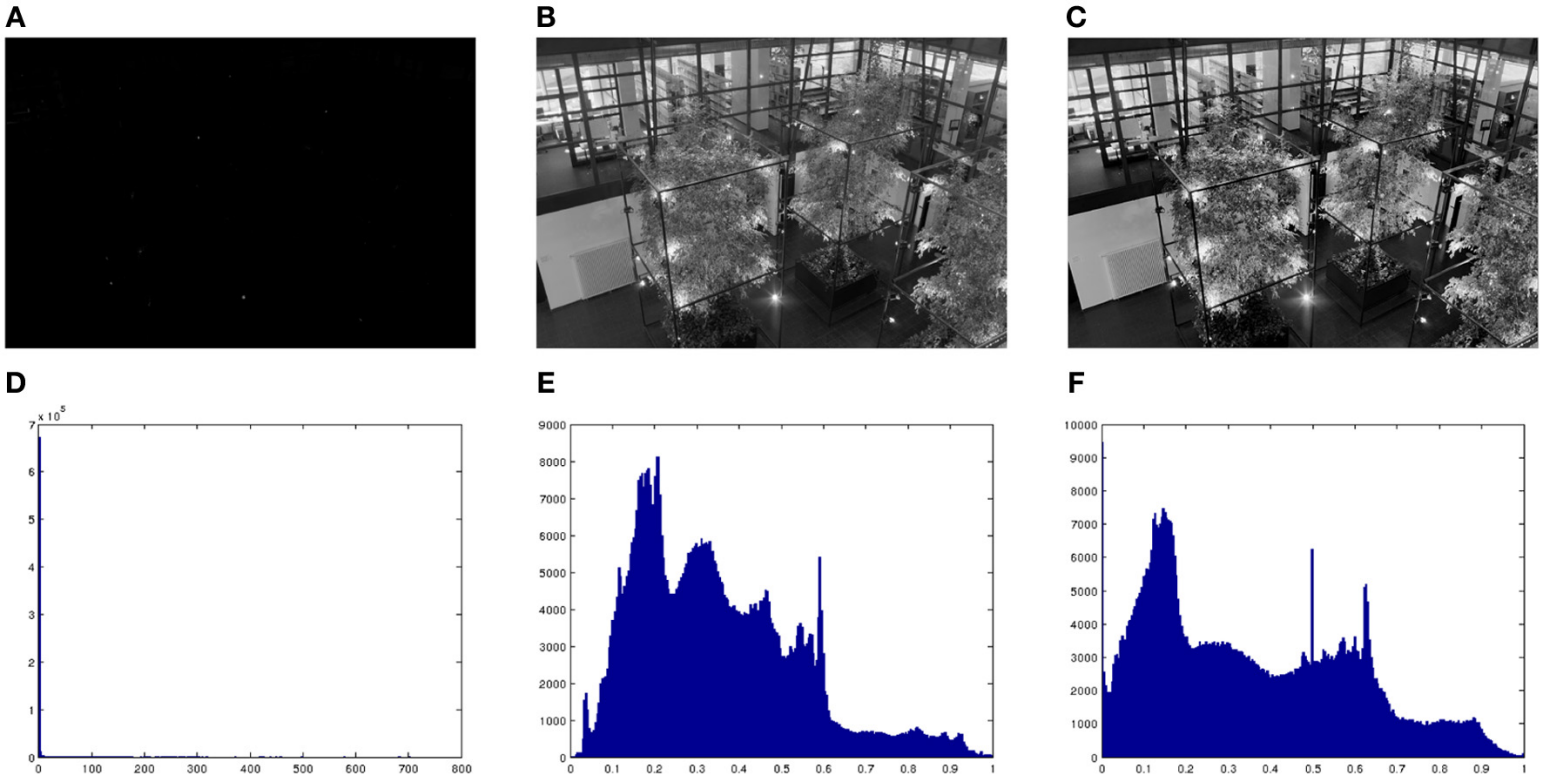
**(A)** High dynamic range (HDR) image, linearly scaled. **(B)** Result of applying the Naka-Rushton equation to the HDR image. **(C)** Result of applying proposed model to image **(B)**. **(D)** Histogram of **(A)**. **(E)** Histogram of **(B)**. **(F)** Histogram of **(C)**. Original image courtesy of Max Planck Institute.

Figure 9A shows a high dynamic range (HDR) image or *radiance map*, linearly scaled to the range [0,1]. Clearly this kind of mapping is useless, which is a way to explain the need for light adaptation and gain control mechanisms in our photoreceptors. Figure 9B shows the result of applying the Naka-Rushton equation to the previous HDR image. Figure 9C shows the result of applying our proposed model to Figure 9B. As we can see, the original radiance image has a very lopsided histogram, which is made considerably more uniform by applying the Naka-Rushton equation and even more flat if we apply our proposed method to the Naka-Rushton output. Local contrast is clearly enhanced as well, see for instance the window frames, the book cases behind the windows, etc. For the implementation details we refer the reader to the Appendix.

**Figure 10:**
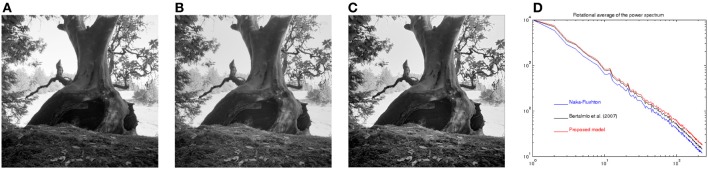
**(A)** Result of applying the Naka-Rushton equation to a high dynamic range image. **(B)** Result of applying the model of Bertalmío et al. (2007) to image **(A)**. **(C)** Result of applying proposed model to image **(A)**. **(D)** Power spectrum of images **(A–C)**. Original image property of Industrial Light and Magic.

Figure 10A shows the result of applying the Naka-Rushton equation to a high dynamic range image. Figure 10B shows the result of applying the model of Bertalmío et al. (2007) to Figure 10A (this is roughly equivalent to the tone mapping approach proposed by Ferradans et al. (2011) in an image processing/computer graphics context). Figure 10C shows the result of applying our proposed model to Figure 10A. Figure 10D compares the power spectrum of the three previous images. We can see that our model improves spectrum whitening over the other two results. In this image the contrast enhancement is more subtle but still noticeable, especially in the interior of the tree-trunk and on the leaves and grass in the foreground.

**Figure 11:**
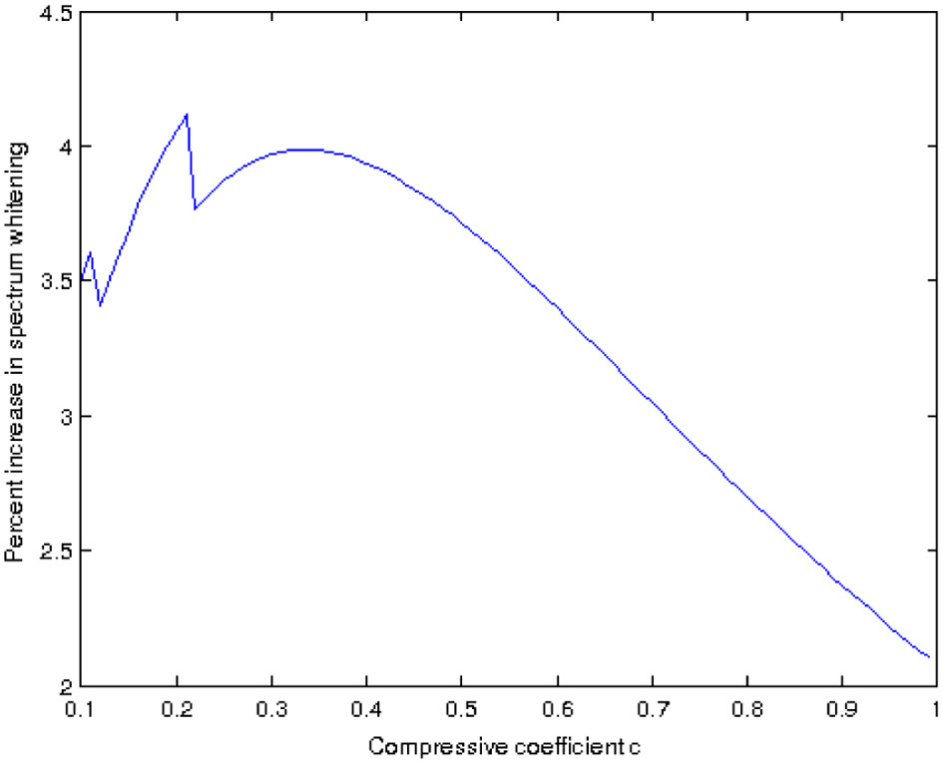
**Increase in spectrum whitening as a function of the constant *c* in Equation 6, for the image in Figure 10**.

An interesting aspect is given by the constant *c* in Equation 6 and its relationship to the whitening of the power spectrum. Given a Naka-Rushton output, we compute the rotational average of its power spectrum which, in log-log coordinates, can be fit by a line with a certain slope. We do the same for the output of our model, that has been applied to the Naka-Rushton output using some value for *c*, and obtain a new linear fit with a new slope. Let us estimate the “increase in whitening” provided by our model as the difference between these two slopes, call it *W*. The value of *W* is a function of the constant *c* used in our model. If we now vary *c* in the interval [0, 1] we can plot the resulting function *W*(*c*), as shown in Figure 11. Disregarding the spikes for low values of *c*, we can see that there is an optimum value for *c*, with which we can obtain the maximum power spectrum whitening that our model can provide. In our model *c* is the power to which we raise the local standard deviation σ(*x*), and this standard deviation is one of the possible measures that are commonly used to estimate local contrast. Because of this reason we are currently investigating the possible connections of our model with the works of Mante et al. (2008) and Kay et al. (2013), since both of them apply a static power-law non-linearity to the contrast. In particular, Kay et al. (2013) computed the value of the exponent of this power law and found that while it varies accross the visual cortex, it is in the range [0, 0.35] with a value of around 1/3 in the case of *V*1: this is all consistent with the tests we have performed so far for different high dynamic range images.

## 5. Conclusion and future work

In this paper we have proposed a neural model, in the form of a Wilson-Cowan equation, that is derived from an image processing technique for local histogram equalization. This new model is able to predict lightness induction phenomena, and improves the efficiency of the representation by flattening both the histogram and the power spectrum of the image signal and increasing local contrast.

We are very much interested in finding evidence of neural responses following our proposed model. Our method performs contrast enhancement, so we would like to explore whether there is any relationship with the work of Martinez et al. (2014), who have very recently confirmed that contrast enhancement takes place at the LGN and is much alike the common techniques used in image processing. Our model has a term where a power law is applied to the contrast, and we can optimize the exponent of this power law so as to maximize the whitening of the spectrum; for the limited tests that we have performed so far, our results appear to be in agreement with what is reported by Mante et al. (2008) and Kay et al. (2013), so we also want to investigate possible connections with those works. And as immediate future work, we will extend our formulation to the color case in order to predict color induction as well.

Last but not least, we believe we can use our proposed model to go back to some image processing and computer vision applications, which could benefit from the insights gained in the visual neuroscience domain. In particular, we are currently working in extending this new model for problems such as tone mapping, gamut mapping and computational color constancy.

### Conflict of interest statement

The author declares that the research was conducted in the absence of any commercial or financial relationships that could be construed as a potential conflict of interest.

## Appendix

In this section we give the implementation details for the results reported in the paper.

For the examples in Figure 6 we computed explicitly the steady state solution of Equation 6, with α = β = γ = 1, the second term of the equation with a weight γ(1 + 5σ^*c*^), and a compression constant of *c* = 1/3 (Figure 6, left) or *c* = 0.75 (Figure 6, right).

For the example in Figure 7 we have adapted the fast numerical implementation of Bertalmío et al. (2007), with a polynomial approximation of degree 7 for the sign function, time step Δ*t* = 0.15 and the stopping condition being fulfilled when the difference between the images of the current and the previous iteration falls below 0.1%. The original image is of size 100 × 200 and the parameter values were: α = β = γ = 1, *c* = 1/3, stencil size for the computation of the standard deviation σ(*x*) : 21 × 21, effective radius of the locality kernel *w*(*x*, *y*) : 75, and of the neighborhood over which we compute the mean average μ(*x*) : 19.

For the examples in Figures 9, 10 we have also used the same fast numerical implementation of Bertalmío et al. (2007), where now the stopping condition is fulfilled when the difference between the images of the current and the previous iteration falls below 0.5%. The parameter values were: α = β = γ = 1, *c* = 1/3, stencil size for the computation of the standard deviation σ(*x*) : 3 × 3, effective radius of the locality kernel *w*(*x*, *y*), effective radius of the neighborhood over which we compute the mean average μ(*x*): 1/3 of the numbers of rows or columns, whichever is larger.
